# Supplementary material for: NREM sleep in the rodent neocortex and hippocampus reflects excitable dynamics
Source: Nat Commun. 2019 Jun 6;10:2478. doi: 10.1038/s41467-019-10327-5 (PMC6554409; doi:10.1038/s41467-019-10327-5)
Supplement: Supplementary file 1 — Supplementary Information [file 41467_2019_10327_MOESM1_ESM.pdf]

Supplementary Information for:

NREM sleep in the rodent neocortex and hippocampus reflects excitable dynamics

*Daniel Levenstein[1,2], György Buzsáki[1,2], John Rinzel[1,3]**

Supplementary Notes

1. *Description of the r-a model and dynamics in the r-a phase plane*
2. *UP/DOWN bistability in the adaptation-free population*
3. *Bifurcation analysis of the model*
4. *Description of the e-i-a model*

Supplementary Discussion

Physiological Interpretation of Model Parameters

General Insight to UP/DOWN dynamics

Supplementary Figures

1. *Slow wave detection*
2. *Recurrence enables UP/DOWN bistability*
3. *Parameter Space of dynamical regimes in the r-a model*
4. *Dynamic regime at the I/O curve center region*
5. *Effective I/O curve for a recurrent adapting population in the presence of noise*
6. *Duration distribution matching method*
7. *Distribution matching in Neocortex and Hippocampus*
8. *Slow variation in cortical state*
9. *Perturbation-evoke slow waves/SWRs*
10. *Bistability and UP/DOWN dynamics in the adapting Inhibition-Stabilized Network*

Supplementary References

[1] Center for Neural Science, New York University

[2] New York University Neuroscience Institute

[3] Courant Institute for Mathematical Sciences, New York University

* Lead Contact, Correspondence: rinzeljm@gmail.com

Supplementary Note 1

Description of the r-a model and dynamics in the r-a phase plane. The r-a model represents the mean firing rate or activity of a neural population with activity-driven adaptation a(t).

$$\tau_{\mathrm{r}}\dot{r} = -r + R_{\infty}\big(wr - ba + I + \xi(t)\big) \qquad (1)$$

$$\tau_{\mathrm{a}}\dot{a} = -a + A_{\infty}(r) \qquad (2)$$

with activation functions

$$R_{\infty}(x) = \frac{1}{1 + e^{-(x - x_0)}}$$

and

$$A_{\infty}(r) = \frac{1}{1 + e^{-k(r - r_0)}}$$

Unless otherwise specified, we use $x_0 = 5$, $r_0 = 0.5 = R_{\infty}(x_0)$, and $k = 15$ to parameterize the activation functions. Values of other parameters are as indicated in the text and figure legends. The rate and adaptation variables can be considered as non-dimensionalized, scaled by their maximum possible values; similarly, we use $\tau_{\mathrm{r}} = 1$ (unless otherwise specified) so that time is dimensionless (AU, arbitrary units), scaled by the time constant for firing rate.

Under constant net input, the population rate will approach a steady state level given by the input-output relation, $R_{\infty}(\mathrm{input})$. Adaptation is similarly activated by neuronal activity to a steady state $A_{\infty}(r)$. For mathematical convenience, $R_{\infty}(\mathrm{input})$ and $A_{\infty}(r)$ are taken to be sigmoidal functions. The time constants τ_{r} and τ_{a} determine how quickly $r(t)$ and $a(t)$ will approach their steady state values and time has been non-dimensionalized to arbitrary model units (AU) so $\tau_{\mathrm{r}} = 1$.

Model dynamics can be represented as a trajectory in the r-a phase plane (Figure 2B, [1]). In the phase plane, trajectories are predictable from Steady states, or fixed points, of activity are found at intersections of the nullclines and may be either stable (attractors) or unstable. The r-nullcline is N-shaped with a right branch at $r{\sim}1$ and a left branch at $r{\sim}0$, which correspond to UP and DOWN states of activity (Figure 2B). When adaptation is slow (i.e. $\tau_{\mathrm{a}} \gg \tau_{\mathrm{r}}$), trajectories move horizontally toward the UP/DOWN branches with time scale τ_r. At the UP/DOWN branches, trajectories drift along the r-nullcline as adaptation activates or inactivates with the time scale τ_{a}. If the branch contains a stable fixed point, the system will remain in the UP or

DOWN state until some perturbation induces a transition to the opposing branch. If there is no fixed point, the trajectory transitions to the opposing branch at the turning point of the r-nullcline (Figure 2B). In this way, an UP or DOWN state in the model can be either stable: requiring a perturbation to evoke a transition to the opposite state, or transient: automatically transitioning to the opposite state due to the activation or inactivation of adaptation.

Supplementary Note 2

UP/DOWN bistability in the adaptation-free population. UP/DOWN alternations are possible only if the population can potentially exist in an UP or a DOWN state. This requires adequate strength of recurrent excitation, w, to self-maintain the UP state under conditions of low drive. We show this first for a reduced case without adaptation dynamics ($b = 0$). In this case, the population rate $r(t)$ satisfies

$$\frac{dr}{dt} = -r + R_\infty(wr + I)$$

The phase space is reduced from a plane to a line, and dynamics of the population rate correspond to motion along the r-axis following eqn. 3 (Supplementary Figure 2A). The motion is rightward (rate increasing) where $dr/dt > 0$ and leftward where $dr/dt < 0$. If recurrent excitation is sufficiently strong, the graph of dr/dt vs. r is N-shaped and there can be two stable (and one unstable) fixed points: an UP state of high activity at $r \approx 1$ and a DOWN state of low activity at $r \approx 0$.

The population rate at fixed points, r_{ss}, depends on the level of drive, I, as is described by the *effective* input/output relation (I/O curve) of the recurrently connected population (Supplementary Figure 2B). If recurrence is weak ($w = 0$), the I/O curve increases monotonically with I. With increased recurrence, the I/O curve shows a central region of bistability between a low-rate fixed point at weak drive and a high-rate fixed point at strong drive. In the I-w parameter space (Supplementary Figure 2C), the bistable region (yellow) has borders that correspond to saddle-node bifurcations at the knees of the I/O curve. UP/DOWN bistability emerges at a critical value of recurrence ($w = 4$), at a level of drive for which the unconnected population would be minimally activated ($I = I_0 - 2$, where $R_\infty(I_0) = 0.5$, see Methods). Consequently, a first general insight of recurrent population rate models is that UP/DOWN bistability will emerge in neuronal populations with sufficiently strong recurrent excitation, during conditions of low drive.

Supplementary Note 3

Bifurcation analysis of the model. With adaptation dynamics reintroduced ($b = 1$), the mechanism for UP/DOWN bistability is like that in the adaptation-free case. Increasing recurrence (larger w) enhances the N-shape of the r-nullcline, allowing for multiple stable fixed points at intersections of the r- and a-nullclines (Supplementary Figure 3A). The effective I/O curve is again bistable-centered for strong recurrence (Figure 3A, top). The region of I-w parameter space with multiple fixed points is now “butterfly-shaped” (Supplementary Figure 3D), with a bistable regime again inside the yellow region at higher values of w.

In contrast to the effect of w, stronger adaption (larger b) diminishes the N-shape of the r-nullcline and decreases likelihood for multiple fixed points (Supplementary Figure 3B). As a result, the network can now oscillate at intermediate values of w, for which the I/O curve appears oscillatory-centered (Figure 3A, middle). The oscillatory region in I-w parameter space is blue, with borders corresponding to Hopf bifurcations (Supplementary Figure 3D). Increasing the strength of adaptation increases the domain of this oscillatory region in I-w parameter space (Supplementary Figure 3F).

Increasing drive raises the r-nullcline and brings the population from a stable DOWN state at low drive to a stable UP state at high drive, with fixed points that trace out the I/O curve (Supplementary Figure 3C). The effective I/O relations, accounting for noise, are represented by statistical properties of UP/DOWN state durations (Figure 3C,D). The duration distributions plotted vs. drive form a crossed-pair, with a center symmetrical portion (i.e. an oscillatory (Figure 3C) or bistable (Figure 3D) regime) flanked by the asymmetrical $\text{Excitable}_{\text{DOWN}}$ and $\text{Excitable}_{\text{UP}}$ regimes. A complementary view of the I/O properties is the graph of decreasing fraction of time in the DOWN state as drive is increased. This Prob(DOWN), or silence density, has been used as an experimental metric of the degree of cortical synchronization [2]. By this terminology, more synchronized regimes correspond to more time spent in the DOWN state, and less synchronized regimes correspond to more time spent in the UP state. Increased drive eventually leads to an UP-only (asynchronous) regime. Thus, the level of drive defines a spectrum from more synchronized to less synchronized dynamics in the model defines a set of characteristic axes in the “space” of UP/DOWN alternation dynamics.

We next describe an analysis of the dynamic regime at the I/O curve's center region, which reveals how the relative strength of recurrence and adaptation form a spectrum from bistable to oscillatory dynamics (Figure 3B).

To analyze the parameter space of the model, we used standard procedures from dynamical systems theory (Strogatz). To determine the linear stability and class of a fixed point ($\dot{r} = \dot{a} = 0$) at $[r^*,\ a^*]$, we evaluate the eigenvalues of the Jacobian matrix

$$J = \begin{bmatrix} \frac{\partial \dot{r}}{\partial r} & \frac{\partial \dot{r}}{\partial a} \\ \frac{\partial \dot{a}}{\partial r} & \frac{\partial \dot{a}}{\partial a} \end{bmatrix}_{[r^*\ a^*]} = \begin{bmatrix} -1 + \frac{\partial R_\infty(X)}{\partial r} & \frac{\partial R_\infty(X)}{\partial a} \\ \frac{1}{\tau}\frac{\partial A_\infty(r)}{\partial r} & -\frac{1}{\tau} \end{bmatrix}_{[r^*\ a^*]}$$

where $X = wr - ba + I$ is the total input to the population. Simplifying the partial derivatives as

$$\frac{\partial R_\infty(X)}{\partial r} = \frac{\partial R_\infty(X)}{\partial X}\frac{\partial X}{\partial r} = w\frac{\partial R_\infty(X)}{\partial X}$$

$$\frac{\partial R_\infty(X)}{\partial a} = \frac{\partial R_\infty(X)}{\partial X}\frac{\partial X}{\partial a} = -b\frac{\partial R_\infty(X)}{\partial X}$$

gives the Jacobian for an arbitrary fixed point:

$$J = \begin{bmatrix} -1 + w\frac{\partial R_\infty(X)}{\partial X} & -b\frac{\partial R_\infty(X)}{\partial X} \\ \frac{1}{\tau}\frac{\partial A_\infty(r)}{\partial r} & -\frac{1}{\tau} \end{bmatrix}_{[r^*\ a^*]}$$

where

$$\frac{\partial R_\infty(X)}{\partial X} = \frac{e^{-(X-x0)}}{(e^{-(X-x0)}+1)^2} = R_\infty(X)(1 - R_\infty(X))$$

$$\frac{\partial A_\infty(r)}{\partial r} = \frac{ke^{-k(r-r0)}}{(e^{-k(r-r0)}+1)^2} = kA_\infty(r)(1 - A_\infty(r))$$

and thus, at steady state

$$J = \begin{bmatrix} -1 + wr(1-r) & -br(1-r) \\ \frac{1}{\tau}a(1-a) & -\frac{1}{\tau} \end{bmatrix}$$

We define $I_{1/2}$ as the level of drive for which there is a fixed point at $r = a = 0.5$, and thus

$$r = R_\infty(wr - ba + I)$$

$$0.5 = R_\infty\big(w(0.5) - b(0.5) + I_{1/2}\big)$$

$$I_{1/2} = R_\infty^{-1}(0.5) + 0.5(b - w)$$

$$I_{1/2} = x_0 - 0.5(w - b)$$

When there is sufficient recurrent excitation for UP/DOWN alternations (i.e. $w > w_0$, see

derivation of w_0 below), $I_{1/2}$ gives the level of drive for equi-duration UP and DOWN states, for a given level of recurrence and adaptation strength (Figure S3).

If we define $I^* = I - I_{1/2}$ as the drive relative to $I_{1/2}$, the bifurcation diagram in I^*-w parameter space (Supplementary Figure 4B) reveals that $I^* = 0$ (i.e. $I = I_{1/2}$) acts as an axis of symmetry of the effective I/O curve, with Excitable$_{\text{DOWN/UP}}$ regimes surrounding a bistable or oscillatory regime that has equi-duration UP/DOWN states at $I^* = 0$, depending on the values of w, b. Furthermore, transitions of the dynamic regime at the center of the I/O curve happen at $I^* = 0$. Supplementary Figure 4C shows the bifurcations at $I^* = 0$ with changing w and fixed $b = 1$. As w is increased, the fixed point at $r = 0.5$ loses stability with the appearance of oscillations in a Hopf bifurcation at $w = w_0$. With further increased values of w, two stable fixed points appear at high and low rate, marking the transition from oscillations to bistability in a pair of saddle node bifurcations at $w = w_\chi$. Finally, the pair of “inner” unstable fixed points coalesce in a pitchfork bifurcation at $w = w_{\mathrm{PF}}$. Thus, the bifurcations at $I^* = 0$ reveal the parameter values at which qualitative changes in the I/O curve occur, between monotonic stable, oscillatory-centered, and bistable-centered I/O curves (Figure 3B).

To solve for the type of fixed point at $I = I_{1/2}$, we use the total input

$$X = wr - ba + I_{1/2}$$

$$X = w(r - 0.5) - b(a - 0.5) + x_0$$

For the fixed point at $[r^*\ a^*] = [0.5\ 0.5]$, $X = x_0$, giving

$$J = \begin{bmatrix} -1 + \frac{w}{4} & -\frac{b}{4} \\ \frac{k}{4\tau} & -\frac{1}{\tau} \end{bmatrix}$$

which we can use to obtain the conditions for w_0 and w_{PF}.

The condition for a Hopf bifurcation at w_0 is that J has a pair of purely imaginary eigenvalues $\lambda_\pm = 0 \pm bi$ (Strogatz). $\lambda_\pm$ can be found by

$$\lambda_\pm = \frac{1}{2}\left(\mathrm{Tr} \pm \sqrt{\mathrm{Tr}^2 - 4\mathrm{Det}}\right)$$

where Tr and Det are the trace and determinant of J, respectively. The value for w_0 is found from the null-trace condition:

$$\mathrm{Tr} = 0$$

$$\left(-1 + \frac{w_0}{4}\right) + \frac{-1}{\tau} = 0$$

$$w_0 = 4\left(1 + \frac{1}{\tau}\right)$$

and minimal value for b follows from:

$$0 < \ \mathrm{Det}$$

$$0 < \left(-1 + \frac{w_0}{4}\right)\left(\frac{-1}{\tau}\right) - \left(\frac{b}{4}\right)\left(\frac{k}{4\tau}\right)$$

$$b > \frac{16}{\tau k}$$

The pitchfork bifurcation at w_{PF} is the transition from 5 (3 unstable) to 3 (1 unstable) fixed points. w_{PF} satisfies the condition that J has a degenerate pair of eigenvalues $\lambda_{\pm} = 0$.

$$0 = \frac{1}{2}\left(\mathrm{Tr} \pm \sqrt{\mathrm{Tr}^2 - 4\mathrm{Det}}\right)$$

$$0 = \mathrm{Det}$$

$$0 = \left(-1 + \frac{w_{\mathrm{PF}}}{4}\right)\left(\frac{-1}{\tau}\right) - \left(\frac{b}{4}\right)\left(\frac{k}{4\tau}\right)$$

$$w_{\mathrm{PF}} = \frac{bk}{4} + 4$$

The degenerate pair of saddle node bifurcations at w_{χ}, which separates oscillatory-centered and bistable-centered I/O curves, is determined numerically using XPP.

Together, this analysis reveals the shape of the I/O curve in the w-b parameter space. For low levels of recurrence, the I/O curve increases monotonically with a stable fixed point for each I-value and no UP/DOWN alternations are possible. At a critical value of recurrence, UP/DOWN alternations emerge at the axis of symmetry of the I/O curve. When adaptation is very weak, only bistability is possible and UP/DOWN alternations emerge in a cusp bifurcation as in Supplementary Figure 2C. With sufficient adaption UP/DOWN alternations emerge in Hopf bifurcation as in Supplementary Figure 3D. With sufficient recurrent excitation, the population will have a bistable-centered I/O curve if recurrence is stronger (Figure 3B, yellow) or an oscillatory-centered I/O curve if adaptation is stronger (Figure 3B, blue). Thus, the relative strength of recurrence and adaptation defines a spectrum from bistable-centered to oscillatory-centered response properties in the model.

Supplementary Note 4

Description of the E-I-A model. Our two-variable model captured significant features of UP/DOWN alternations and the relative roles of adaptation and recurrent excitation. However,

firing rate in the UP state was described only as “active”, limited by the saturating input-output function $R_\infty(\mathrm{input})$. On the other hand, neuronal spike rates during the UP state are generally low due to balanced excitation and inhibition. The e-i-a model represents the mean rate of an adapting excitatory and an inhibitory population,

$$\tau_\mathrm{e}\dot{r}_\mathrm{e} = -r_\mathrm{e} + R_{\mathrm{e},\infty}\big(w_\mathrm{ee}r_\mathrm{e} - w_\mathrm{ei}r_\mathrm{i} - ba + I_\mathrm{e} + \xi_\mathrm{e}(t)\big) \qquad (3)$$

$$\tau_\mathrm{i}\dot{r}_\mathrm{i} = -r_\mathrm{i} + R_{\mathrm{i},\infty}\big(w_\mathrm{ie}r_\mathrm{e} - w_\mathrm{ii}r_\mathrm{i} + I_\mathrm{i} + \xi_\mathrm{i}(t)\big) \qquad (4)$$

$$\tau_\mathrm{a}\dot{a} = -a + A_\infty(r_\mathrm{e}) \qquad (5)$$

with power law activation functions, as in Ahmadian and Milller (2013)[29].

$$R_{\mathrm{e/i},\infty}(x) = k[x]_+{}^n$$

The activation function of adaptation, $A_\infty(r)$, is the same as in the r-a model, with parameters $r_0 = 2$, and $k = 3$. Unless otherwise specified, we’ve assumed for simplicity $w_\mathrm{ee} = w_\mathrm{ie} = w_\mathrm{e}$ and $w_\mathrm{ii} = 0$. However, the behaviors are robust to a range of weight values.

Supplementary Discussion

Physiological interpretation of model parameters. Our model (Equations (1,2)) describes the mean activity of a neuronal population with positive feedback from recurrent excitation (wr), slow negative feedback from adaptation (ba), and a source of noisy drive ($I + \xi(t)$). We interpret drive in the model as the combination of external input and various other factors that drive cells toward spiking, including internal (for example, miniEPSPs) and modulatory influences (for example, increasing the excitability of cells would correspond to an increase in drive parameter in our model). The recurrence parameter, w, reflects the effective weight of excitatory synapses within the population. This self-excitation drives the population during the UP state. In the two-variable model, the saturating I/O relation, $R_\infty(\text{input})$, imposes a maximum activity at $r(t) = 1$. However, the three-variable model reveals that fast negative feedback from inhibitory cells can dynamically stabilize the rate of excitatory population during the UP state.

Adaptation could encompass a variety of physiological processes that are activated by, and subsequently reduce, spiking at a slow timescale (~50ms-1s), such as slow voltage or calcium activated potassium currents, or sodium current inactivation [3,4]. For mathematical simplicity, we have reduced the effect of adaptive processes, to a single, saturating, mean field variable, $a(t)$, that negatively feeds back on population activity with strength b. While we have used a sigmoidal activation function for adaptation, this decision is not crucial for the dynamics described. With a linear activation function we would have been able to get similar results (albeit without the possibility for 5-Fixed point regimes). The choice of a sigmoid activation function was made for two reasons: 1) we found that sigmoid adaptation increases the robustness of the excitable regimes – it decreases the noise required for transitions out of stable fixed points and extends the parameter domain in which excitable alternations are seen. 2) Biologically, adaptation would be expected to saturate; for example, if adaptation were due to a voltage-gated ionic current. Previous studies have also modeled synaptic or slow divisive feedback to achieve the same goal [5,6], in some cases showing characteristic differences [5]. Further work to identify signatures of different sources of adaptive feedback could give insight to the implications and identification of biophysical substrates of adaptation in different physiological contexts.

With these idealizations, our model encompasses numerous previous models for UP/DOWN dynamics, from mean field to large scale spiking models. We have reduced the critical influences to a few key parameters that provide an intuitive understanding of a wide range of UP/DOWN alternation dynamics in neuronal populations.

General insight to synchronized UP/DOWN dynamics. Experiments in multiple physiological contexts have revealed that SWRs and UP/DOWN alternations are a locally-generated “default state” of hippocampal and neocortical tissue [7,8], ubiquitous under conditions of low neuromodulatory tone. Beyond NREM, they are observed during quiet wakefulness, [9,10] under anesthesia [11,12], and during *in vitro* slice or culture preparations [13,14] and in isolated tissue preprations [15,16]. Our model reveals why UP/DOWN alternations are so ubiquitous: they are seen in neural populations with sufficiently strong local recurrent excitation (>w_0) at comparatively low levels of drive $(\sim I_{1/2})$.

Synchronized UP/DOWN dynamics have been proposed to exist in a “multi-dimensional spectrum” [17]. Our model captures a subspace of this spectrum defined by two axes (Figure 7A). First, the magnitude of excitatory drive determines the degree of synchronization [2]. Increasing drive brings the population from a DOWN-dominated “more synchronized” regime with brief population bursts, to an UP-dominated “more asynchronous” regime with occasional DOWN states. The relative strength of recurrent excitation and adaptation determines the temporal dynamics between these extremes. When recurrence dominates, the system is characterized by bistability; when adaptation dominates, oscillations emerge. We note that this second axis also influences the “steepness” of UP/DOWN transitions, even in the adjacent excitable regimes, which we have not explored.

Different experimental conditions are associated with different regimes in the spectrum of UP/DOWN dynamics captured by our model. Other models have described oscillatory UP/DOWN dynamics in the spinal cord [18] and in cortical slices [19], whereas in cortical cultures Excitable$_{\mathrm{DOWN}}$-like bursting behavior dominates [20]. Recently, bistable UP/DOWN dynamics were found to describe the activity of sensory cortex during urethane anesthesia [2,21]. During quiet wakefulness, cortical state varies following the level of arousal of the animal [22], but is unclear which of the regimes. Our model provides a framework by which one can predict how duration statistics should change with experimental manipulation of intrinsic or network properties, for example by varying levels of anesthesia or applying other pharmacological agents.

Extensive study has revealed multiple factors that can bring cortical tissue from the “default mode” of UP/DOWN dynamics to an activated state. In culture and slice preparations, increased levels of subcortical neuromodulators are able to ‘wake up’ the tissue, leading to the replacement of slow oscillations in the neocortex with asynchronous spiking [20] and SWRs in the hippocampus with theta-like oscillations [23]. Acetylcholine induces neocortical desynchronization

and hippocampal theta during REM sleep[24], and acetylcholine, norepinephrine, or thalamic drive can give rise to cortical desynchronization during quiet wakefulness [25,26]. Each of these factors have effects that correspond to parameter changes that can transition our model from UP/DOWN dynamics to an asynchronous (tonic UP) state: 1) increasing excitability or external drive, I, 2) decreasing recurrence below the critical value for UP/DOWN alternations, $w < w_0$, or 3) decreasing the strength of adaptation, b, or 4) increasing recurrence such that the DOWN state loses stability. Similarly, the ascending neuromodulators increase excitability by depolarizing cortical pyramidal cells, decrease the effective weight of excitatory-excitatory synapses, and deactivate adaptive currents [27,28]. By these parallels, we are able to apply our model to interpret the putative mechanisms by which cortical desynchronization follows global and local levels of arousal.

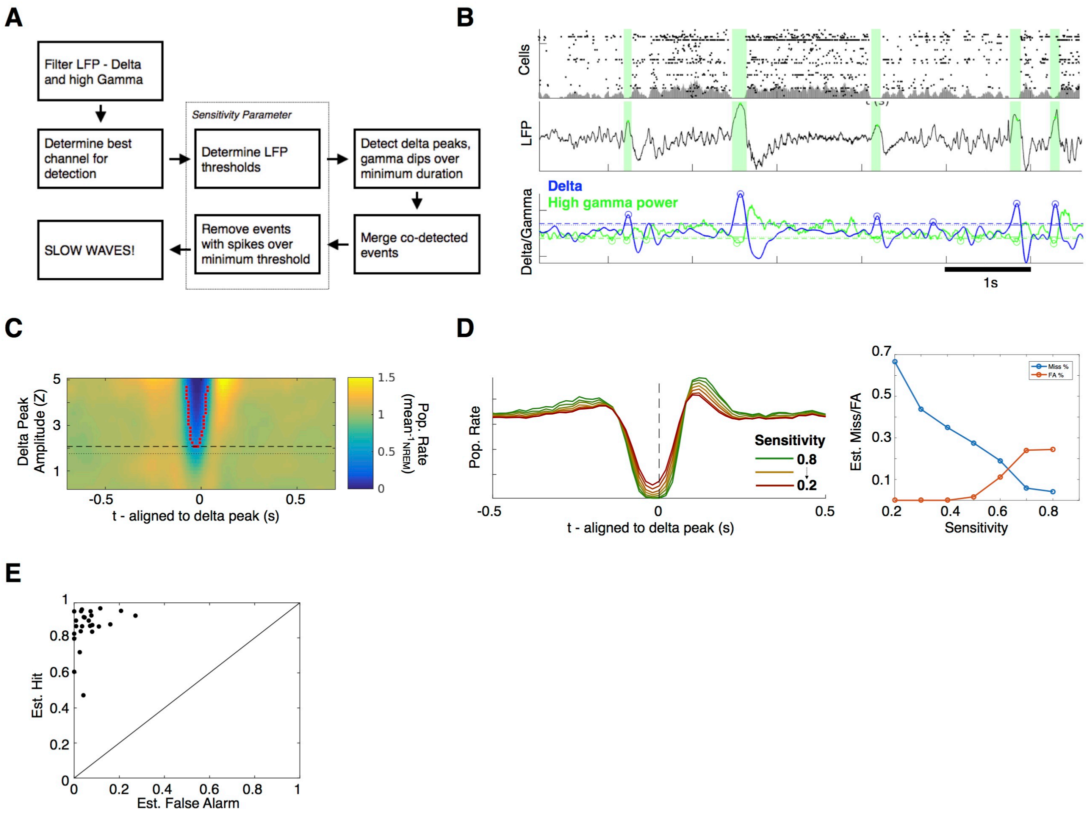

Supplemental Figure 1 Slow wave detection. A: The process for slow wave detection, as described in the Methods, is schematized. **B:** A 7s sample of neocortical data is shown with spike raster (top), LFP (middle), and filtered delta and high gamma power (bottom). Detection thresholds, as determined in panel C, are indicated as dashed (peak) and dotted (window) lines. Slow waves are detected as coincidence of threshold crossings of both signals. **C:** Threshold detection. Slow wave threshold was determined by calculating the population PETH around delta peaks, as a function of peak amplitude. Peak threshold (dashed line) was taken to be the lowest amplitude for which the PETH drops below a (mean-normalized) rate (given as the "sensitivity parameter"). Window threshold (dotted line) was taken to be the average value of the delta signal at which the PETH drops below the sensitivity parameter for any peak value (red dots). **D:** (Left) PETH around detected slow waves in the example recording in B,C for a range of sensitivity parameters. (Right) Estimated miss and false alarm % as a function of sensitivity parameter. Sensitivity = 0.6 was used for this study. **E:** Miss and false alarm % for each recording in the dataset.

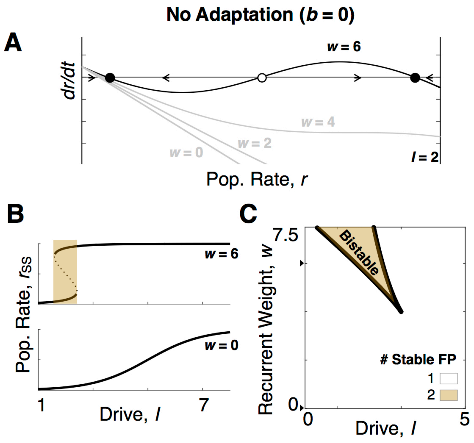

Supplemental Figure 2: Recurrence enables UP/DOWN bistability. A: Geometric analysis of the non-adapting ($b = 0$) model illustrates the mechanism of bistability with sufficient recurrent excitation for fixed $I = 2$. Curve indicates dr/dt for a given value of r. Circles indicate stable (filled) and unstable (empty) fixed points. **B:** Effective population **i**nput-output (I/O) curve, i.e. population rate steady state as a function of drive, for a population with low ($w = 0$) and high ($w = 6$) recurrence. **C:** I-w parameter space, bistable region appears in a cusp bifurcation at high recurrence and low input.

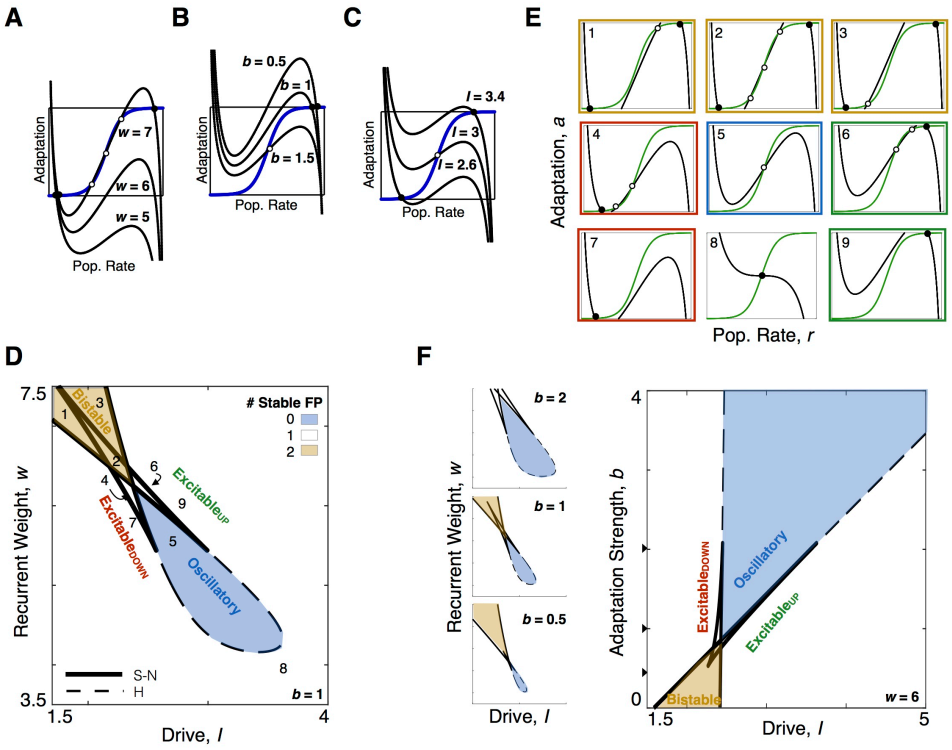

Supplemental Figure 3 Parameter space of dynamical regimes of the *r-a* model.
A: Effect of recurrence on the *r*-nullcline (b = 1, I = 2). **B:** Effect of adaptation strength, b, on *r*-nullcline (w = 6, I = 2.75). **C:** Effect of drive, I, on *r*-nullcline (w = 5, b = 1). **D:** *I-w* Bifurcation diagram reveals the "Butterfly Catastrophe" motif, yellow indicates bistability, blue indicates oscillations. Solid line, saddle-node (S-N) bifurcations; dashed line, Hopf (H) bifurcation. Numbers correspond to the phase planes in panel E. Note that while the 3-fixed point configuration in Figure 2Ciii/iv is limited to the small parameter domain of around number 4/6, excitable dynamics extend out to the 1-fixed point regions, as can be seen in Figure 3D. **E:** Representative phase plane from each domain of parameter space. Regime is determined by the location of stable fixed points. (Yellow: bistable, blue: oscillatory, red: $\text{Excitable}_{\text{DOWN}}$, green: $\text{Excitable}_{\text{UP}}$). **F:** The *I-b* parameter space. Increasing adaptation strength, b, increases the domain of the oscillatory regime. (Left) *I-w* parameter space for different values of b.

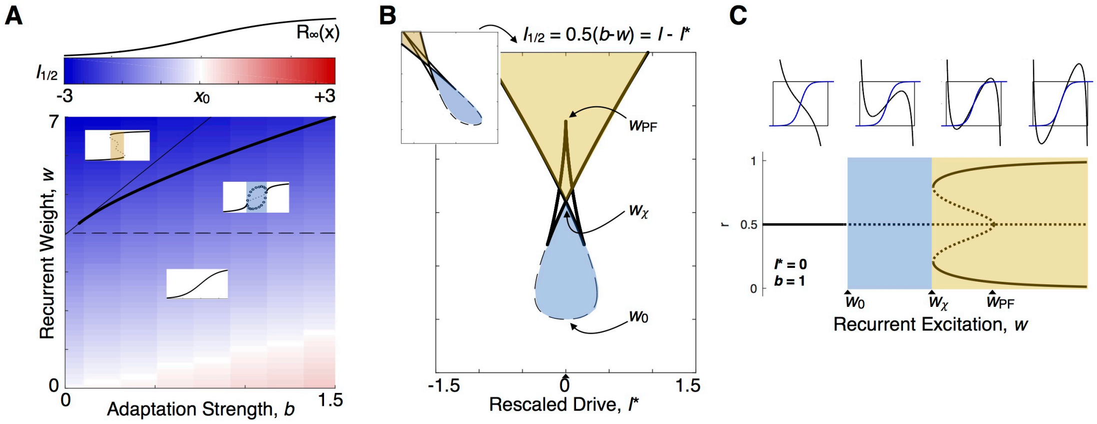

Supplemental Figure 4 Dynamic regime at the I/O curve center region. **A:** The level of drive for half-activation of the effective I/O curve ($I_{1/2}$, see Methods) varies as a function of *w* and *b*. Color indicates $I_{1/2}$ relative to x_0 (the midpoint of the unconnected I/O curve $R_\infty(x)$, shown as reference). Lines reflect bifurcations of the effective I/O curve, as shown in Figure 3A, revealing that the operating range for *I* shifts to lower values (deeper blue) when recurrence is sufficiently high for UP/DOWN alternations. **B:** I^*-*w* parameter space. $I^* = I - I_{1/2}$ centers the parameter space around the I/O curve. **C:** Bifurcations in *w*, with $I^* = 0$ (i.e. at $I = I_{1/2}$). Oscillations appear in a hopf bifurcation at w_0, two stable (and two unstable) fixed points appear in a pair of saddle-node bifurcations at w_χ, and the unstable fixed points coalesce with the center unstable fixed point in a pitchfork bifurcation at w_{PF}. Each of these bifurcations is shown in the *w-b* plane in Figure 3A.

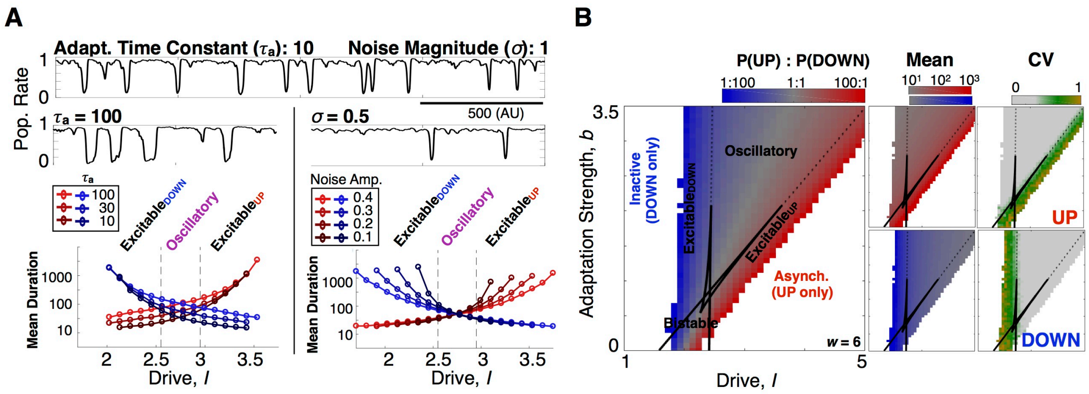

Supplemental Figure 5 Effective I/O curve for a recurrent adapting population in the presence of noise. A: Distinct effect of time scale of adaptation (τ_a) and noise magnitude (σ) on stable and transient states. Increasing the time scale of adaptation increases the duration of transient states. Noise decreases the duration of stable states. **B:** Same as figure 3E, for *I*-*b* parameter space with fixed *w*.

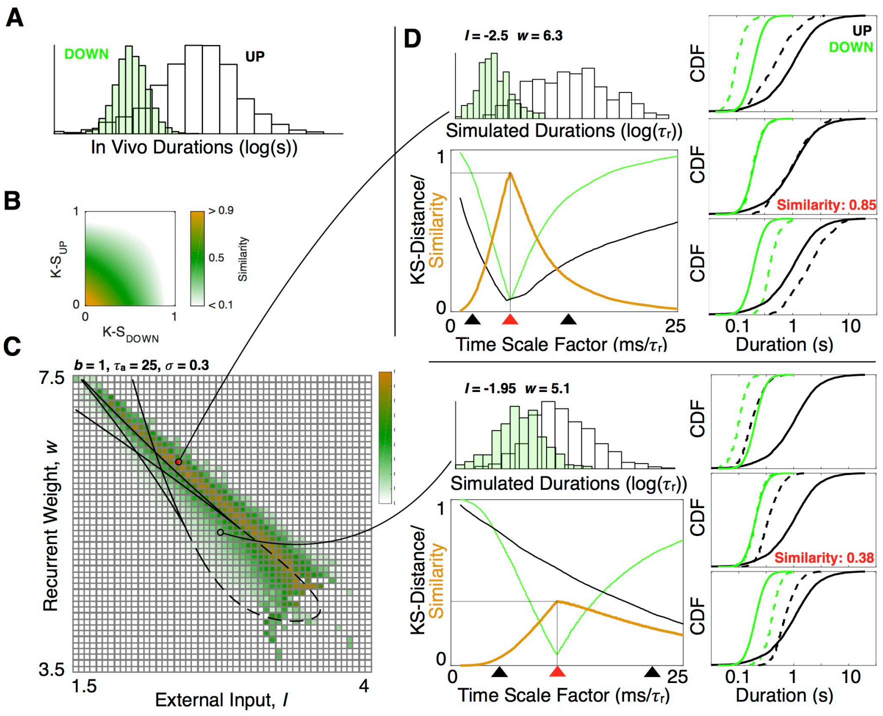

Supplemental Figure 6 Duration distribution matching method. A: UP/DOWN state duration distributions for an example recording. **B:** Similarity as a function of K-S distance between in vivo and simulated UP and DOWN state duration distributions. **C:** Map of similarity between the durations for the example recording in A and the *r-a* model with noise (Figure 3D). **D:** Calculation of similarity for two points in the *I-w* plane. For each point, KS distance between simulated and in vivo UP/DOWN state duration distributions is calculated at time scaling factors between 0.5 and 25ms/τ_r. Simulated (dashed) and in vivo (solid) cumulative distributions are shown for three scaling factors (arrows) at each point. Similarity for the point is taken to be the similarity at the best time scale factor, re-dimensionalizing time to give the best match to the shapes and relative values of the UP and DOWN state duration distributions.

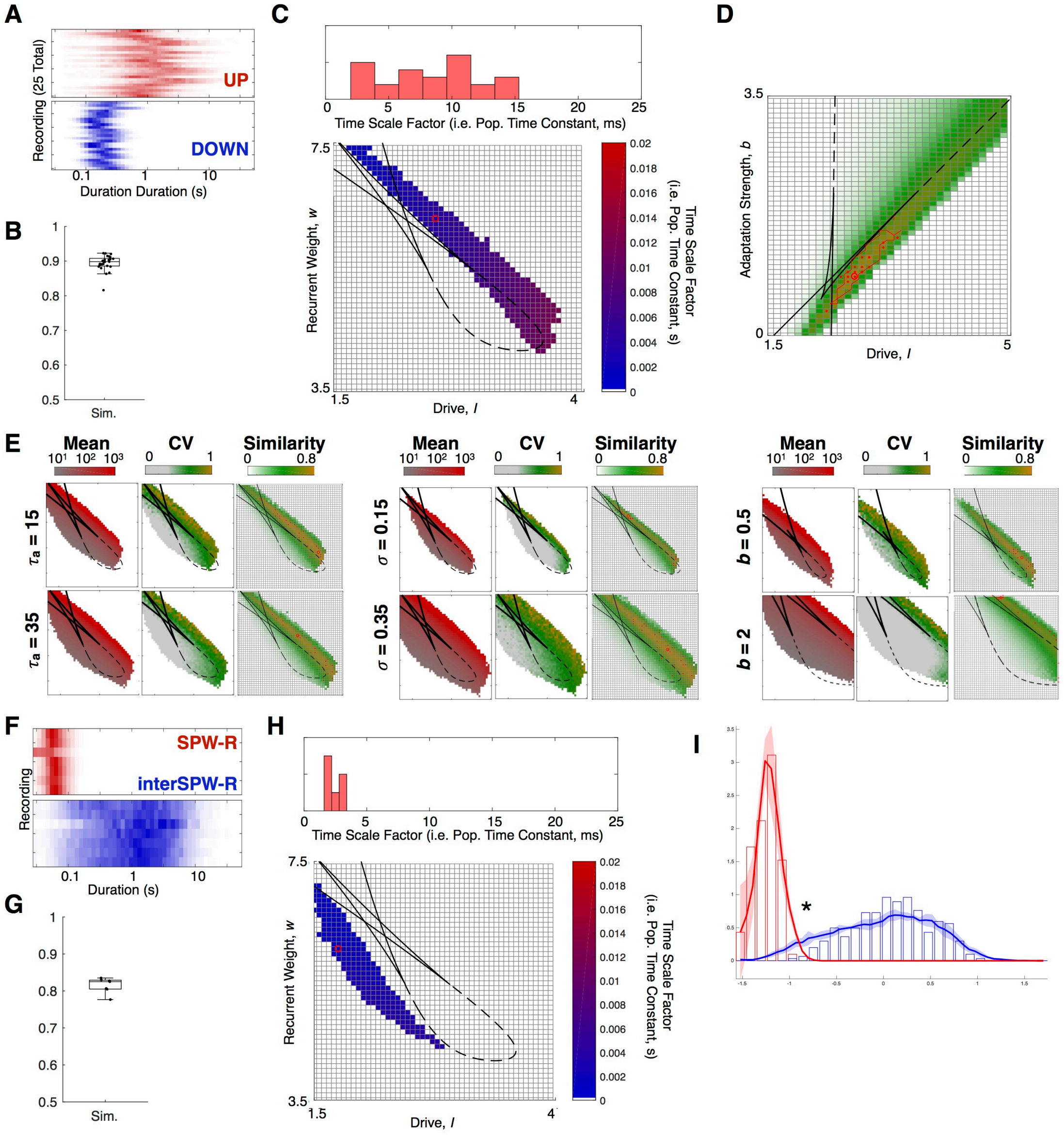

Supplemental Figure 7 Distribution matching in Neocortex and Hippocampus. **A.** UP/DOWN state durations for all neocortical recordings in the dataset. **B:** Similarity metric for each neocortical recording the dataset. **C:** Histogram of time scale factors for all neocortical recordings in the dataset. (Bottom) mean time scale factor in *I-w* parameter space. **D:** Matching in the I-B parameter space. Same as figure 4B. **E:** Mean/CV of UP state durations and data-model similarity in the I-W plane with variation of the fixed parameters: time scale of adaptation, τ_a; magnitude of noise, σ, and adaptation strength, b. All other parameters same as in Figure 4B. **F-H:** Same as A-C, for hippocampus. **I:** Enlarged HPC duration distributions - model does not capture short duration interSWR periods.

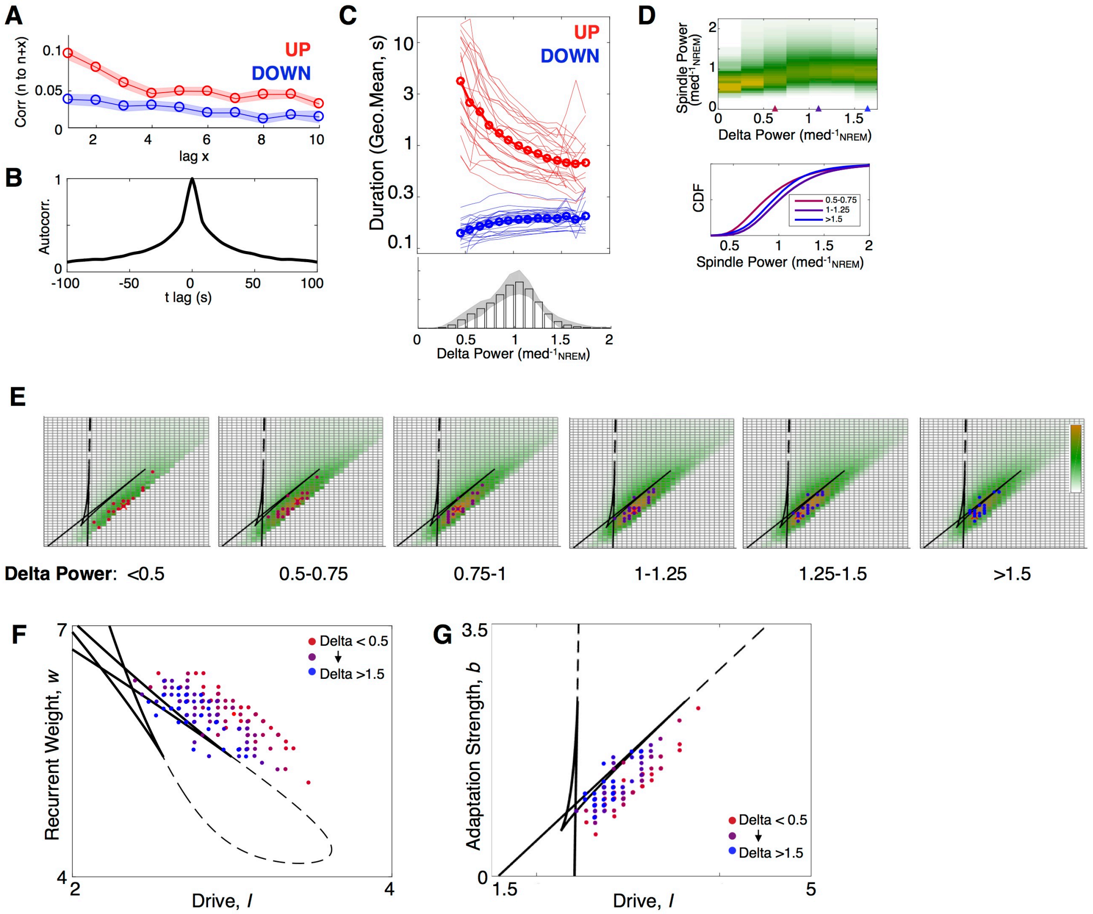

Supplemental Figure 8 Slow variation in cortical state. **A:** Correlation between durations of adjacent UP/DOWN states. **B:** Autocorrelation of delta power. **C:** Mean UP/DOWN state duration plotted vs delta power. Light lines: geometric mean duration for each recording. Dark lines: geometric mean duration over all recordings. **D:** High spindle power most common for intermediate delta power. Spindle power distribution as a function of delta power over all recordings. (Bottom) cumulative distribution of spindle power for low, intermediate, and high delta power. **E:** Same as Figure 6D but with matching to the *I-b* parameter space. **F,G:** Best matching parameter values in *I-w* and *I-b* parameter space for dwell time distributions in each recording, grouped by delta power (6 groups). Fitting procedure similar to Figure 4, with τ_r fixed at $\tau_r = 5$ms.

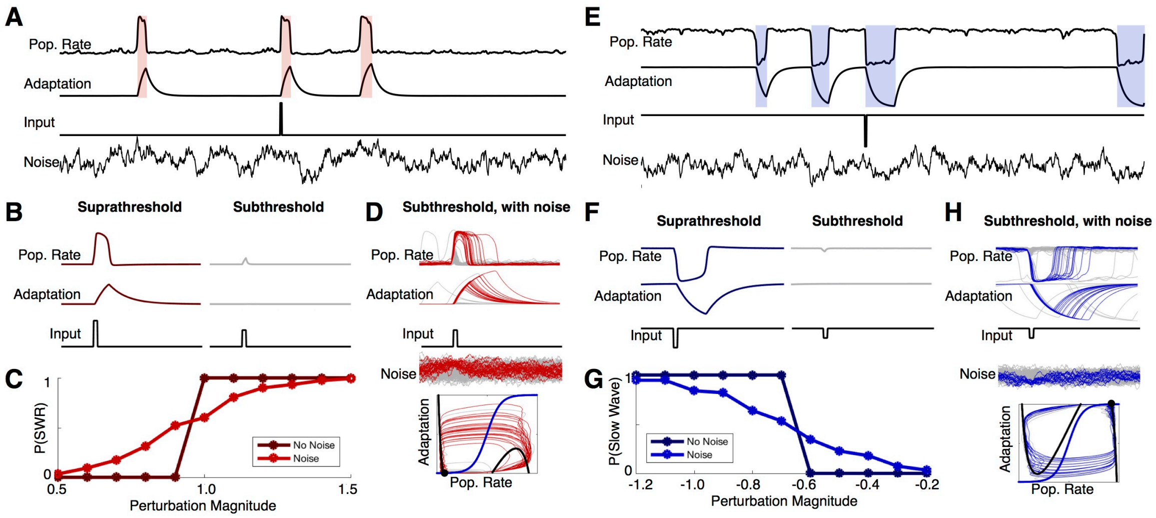

Supplemental Figure 9. Perturbation-evoked Slow Waves/SWRs. A: Simulated r-a model with best-matching parameters to hippocampal SWR dynamics, response to input perturbation ($I_{perturb}$ = 1.25). **B:** Suprathreshold ($I_{perturb}$ = 1.1) and subthreshold ($I_{perturb}$ = 0.7) perturbation of the noise-free model. **C:** Probability of perturbation-evoked SWR as a function of perturbation magnitude, with and without noise (magnitude: 0.2). **D:** Probabilistic SWR response to subthreshold perturbation in the presence of noise - colored trajectories are those in which an UP state was evoked. **E-H:** Same as A-D for the model with best-matching parameters to the cortical slow wave dynamics.

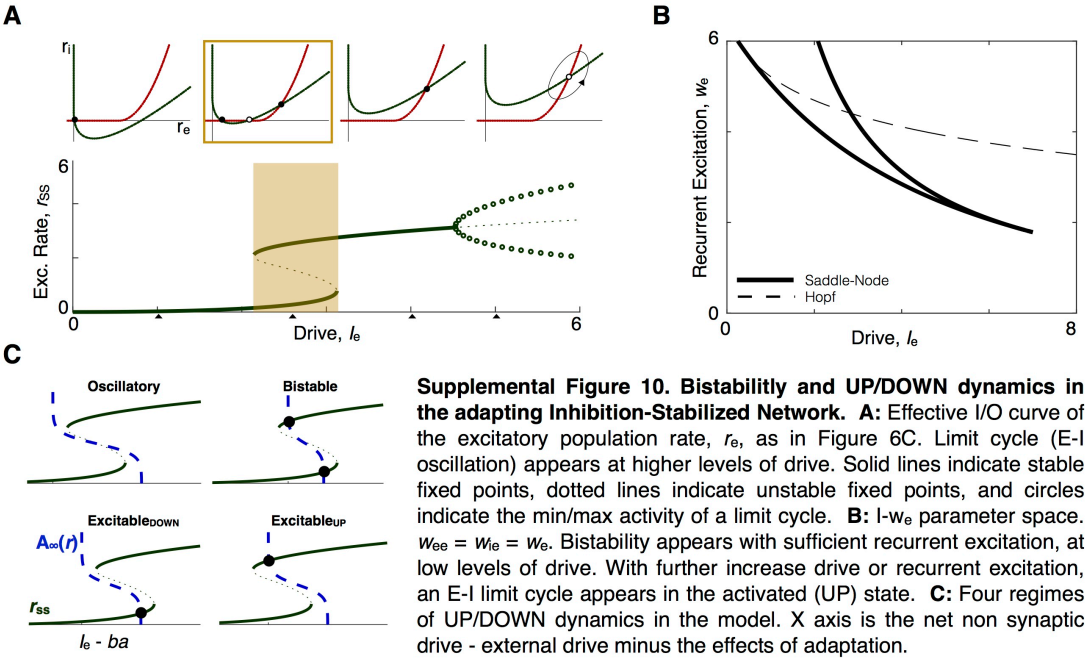

Supplemental Figure 10. Bistabilitly and UP/DOWN dynamics in the adapting Inhibition-Stabilized Network. A: Effective I/O curve of the excitatory population rate, r_e, as in Figure 6C. Limit cycle (E-I oscillation) appears at higher levels of drive. Solid lines indicate stable fixed points, dotted lines indicate unstable fixed points, and circles indicate the min/max activity of a limit cycle. **B:** I-w_e parameter space. $w_{ee} = w_{ie} = w_e$. Bistability appears with sufficient recurrent excitation, at low levels of drive. With further increase drive or recurrent excitation, an E-I limit cycle appears in the activated (UP) state. **C:** Four regimes of UP/DOWN dynamics in the model. X axis is the net non synaptic drive - external drive minus the effects of adaptation.

SUPPLEMENTARY REFERENCES

1. Strogatz, S. H. *Nonlinear dynamics and chaos: with applications to physics, biology, chemistry, and engineering*. (2014).
2. Mochol, G., Hermoso-Mendizabal, A., Sakata, S., Harris, K. D. & la Rocha, de, J. Stochastic transitions into silence cause noise correlations in cortical circuits. *PNAS* **112,** 3529–3534 (2015).
3. Vergara, C., Latorre, R., Marrion, N. V. & Adelman, J. P. Calcium-activated potassium channels. *Curr Opin Neurobiol* **8,** 321–329 (1998).
4. Jung, H. Y., Mickus, T. & Spruston, N. Prolonged sodium channel inactivation contributes to dendritic action potential attenuation in hippocampal pyramidal neurons. *The Journal of Neuroscience* **17,** 6639–6646 (1997).
5. Tabak, J., Rinzel, J. & Bertram, R. Quantifying the relative contributions of divisive and subtractive feedback to rhythm generation. *PLoS Comp Biol* **7,** e1001124 (2011).
6. Holcman, D. & Tsodyks, M. V. The emergence of Up and Down states in cortical networks. *PLoS Comp Biol* **2,** e23 (2006).
7. Buzsáki, G. Rhythms of the Brain. (2006).
8. Sanchez-Vives, M. V., Massimini, M. & Mattia, M. Shaping the Default Activity Pattern of the Cortical Network. *Neuron* **94,** 993–1001 (2017).
9. Petersen, C. C., Hahn, T. T. G., Mehta, M., Grinvald, A. & Sakmann, B. Interaction of sensory responses with spontaneous depolarization in layer 2/3 barrel cortex. *Proc. Natl. Acad. Sci. U.S.A.* **100,** 13638–13643 (2003).
10. Buzsáki, G., Leung, L. W. & Vanderwolf, C. H. Cellular bases of hippocampal EEG in the behaving rat. *Brain Research* **287,** 139–171 (1983).
11. Steriade, M., Nunez, A. & Amzica, F. A novel slow (< 1 Hz) oscillation of neocortical neurons in vivo: depolarizing and hyperpolarizing components. *The Journal of Neuroscience* **13,** 3252–3265 (1993).
12. Ylinen, A. *et al.* Sharp wave-associated high-frequency oscillation (200 Hz) in the intact hippocampus: network and intracellular mechanisms. *The Journal of Neuroscience* **15,** 30–46 (1995).
13. Sanchez-Vives, M. V. & McCormick, D. A. Cellular and network mechanisms of rhythmic recurrent activity in neocortex. *Nature neuroscience* **3,** 1027–1034 (2000).
14. Colgin, L. L., Kubota, D., Jia, Y., Rex, C. S. & Lynch, G. Long-term potentiation is impaired in rat hippocampal slices that produce spontaneous sharp waves. *The Journal of Physiology* **558,** 953–961 (2004).
15. Timofeev, I., Grenier, F., Bazhenov, M., Sejnowski, T. & Steriade, M. Origin of slow cortical oscillations in deafferented cortical slabs. *Cereb. Cortex* **10,** 1185–1199 (2000).
16. Buzsáki, G., Czopf, J., KondÀkor, I., Björklund, A. & Gage, F. H. Cellular activity of intracerebrally transplanted fetal hippocampus during behavior. *Neuroscience* **22,** 871–883 (1987).
17. Harris, K. D. & Thiele, A. Cortical state and attention. *Nat Rev Neurosci* **12,** 509–523 (2011).
18. Vladimirski, B. B., Tabak, J., O'Donovan, M. J. & Rinzel, J. Episodic activity in a heterogeneous excitatory network, from spiking neurons to mean field. *J Comput Neurosci* **25,** 39–63 (2008).
19. Mattia, M. & Sanchez-Vives, M. V. Exploring the spectrum of dynamical regimes and

timescales in spontaneous cortical activity. *Cogn Neurodyn* **6,** 239–250 (2012).
20. Hinard, V. *et al.* Key electrophysiological, molecular, and metabolic signatures of sleep and wakefulness revealed in primary cortical cultures. *Journal of Neuroscience* **32,** 12506–12517 (2012).
21. Jercog, D. *et al.* UP-DOWN cortical dynamics reflect state transitions in a bistable network. *eLife* (2017).
22. McGinley, M. J., David, S. V. & McCormick, D. A. Cortical Membrane Potential Signature of Optimal States for Sensory Signal Detection. *Neuron* **87,** 179–192 (2015).
23. Konopacki, J., Maciver, M. B., Bland, B. H. & Roth, S. H. Theta in hippocampal slices: relation to synaptic responses of dentate neurons. *Brain Research Bulletin* **18,** 25–27 (1987).
24. Jouvet, M. *The Paradox of Sleep: The Story of Dreaming. Translated by Laurence Garey (1999)*. (1994).
25. Eggermann, E., Kremer, Y., Crochet, S. & Petersen, C. C. Cholinergic Signals in Mouse Barrel Cortex during Active Whisker Sensing. *Cell Rep* **9,** 1654–1660 (2014).
26. Poulet, J. & Petersen, C. C. Internal brain state regulates membrane potential synchrony in barrel cortex of behaving mice. *Nature* **454,** 881–885 (2008).
27. Cole, A. E. & Nicoll, R. A. Acetylcholine mediates a slow synaptic potential in hippocampal pyramidal cells. *Science* **221,** 1299–1301 (1983).
28. Hasselmo, M. E. & McGaughy, J. High acetylcholine levels set circuit dynamics for attention and encoding and low acetylcholine levels set dynamics for consolidation. *Prog. Brain Res.* **145,** 207–231 (2004).
29. Ahmadian, Y., Rubin, D. B. & Miller, K. D. Analysis of the stabilized supralinear network. *Neural Computation* **25,** 1994–2037 (2013).
